# HIV/AIDS mortality in a south east European country versus a west European country

**DOI:** 10.7448/IAS.17.4.19620

**Published:** 2014-11-02

**Authors:** Gordana Dragovic, Colette Smith, Djordje Jevtovic, Jovana Kusic, Dubravka Salemovic, Jovan Ranin

**Affiliations:** 1 Department of Pharmacology and Clinical Pharmacology, School of Medicine, University of Belgrade, Belgrade, Serbia and Montenegro; 2 Research Department of Infection and Population H, UCL Medical School, London, UK; 3 School of Medicine, University of Belgrade and Infectious and Tropical Diseases Hospital, Belgrade, Serbia and Montenegro

## Abstract

**Introduction:**

Antiretroviral (ARV) treatment available in low-middle income countries differs as suggested in international HIV-treatment guidelines. Thus, we compared ARV regimens introduced as a first-line therapy, time of initiation, frequency of making combination antiretroviral therapy (cART) switches, frequency of viral and immunological monitoring and treatment outcome in south east European (SEE) country (i.e. HIV Centre in Belgrade, Serbia, (HCB)) and west European country (i.e. Royal Free Centre for HIV Medicine at the Royal Free Hospital London, UK (RFH)).

**Materials and Methods:**

ARV naïve patients starting cART from 2003 to 2012 were included. Comparisons of the two cohorts were made using a chi-square test or Fisher's exact test for categorical variables and a Mann-Witney U test for continuous variables. Kaplan Meier survival curves were compared using the log rank test.

**Results:**

Of 597 patients from HCB, 361 (61%) initiated cART with prior AIDS diagnosed, while 337 (19%) of 1763 patients from RFH. Average baseline CD4+ T cell counts were significantly lower in Serbia than in UK (177 cells/mm^3^ vs 238 cells/mm^3^). The total (mediana, IQR) CD4+ T cell count measurements in the first year of cART was 2 (1, 2) at the HCB, while it was statistically significant higher at the RFH 5 (3, 7), respectively (p<0.0001). At the RFH, it appeared that the cART switching is due to patient's preference or toxicity (46%), while the lack of supply and toxicity (37%) were the most important reasons for treatment change in HCB, within the same period of time (p<0.05). Mortality rates were higher at the HCB versus RFH (p<0.0001). After 12, 24 and 36 months of cART, 3%, 5% and 8% of patients died in HCB, whereas 2%, 3% and 4% of patients died in RFH, respectively (Figure 1).

**Conclusions:**

In south European countries, as a consequence of low testing rate, ARV treatment is introduced at an advanced stage of disease, having a high mortality rate as a consequence. Switching within ARV drugs appears often due to lack of drug supplies and frequently drug-related toxicity in south east Europe, while in the east European country due to patient's preferences and rarely due to drug-related toxicity.

**Figure 1:**
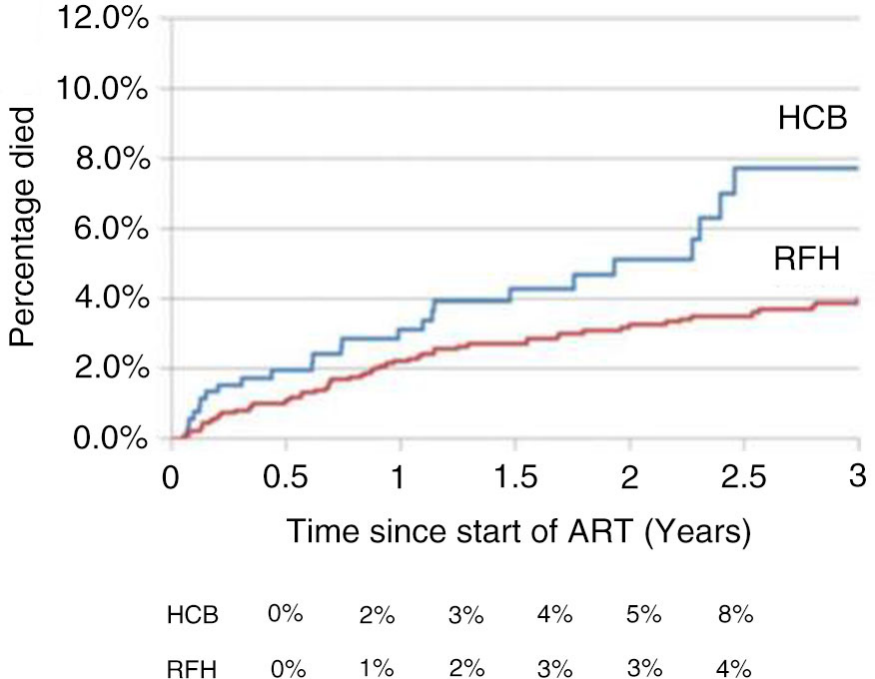
Mortality in HCB and RFH after 3 years of introducing cART.

